# Treatment stage migration and treatment sequences in patients with hepatocellular carcinoma: drawbacks and opportunities

**DOI:** 10.1007/s00432-021-03528-3

**Published:** 2021-02-04

**Authors:** Cyrill Wehling, Michael T. Dill, Alexander Olkus, Christoph Springfeld, De-Hua Chang, Patrick Naumann, Thomas Longerich, Clemens Kratochwil, Arianeb Mehrabi, Uta Merle, Jan Pfeiffenberger, Christian Rupp, Karl Heinz Weiss, Markus Mieth

**Affiliations:** 1 Department of Gastroenterology and Hepatology, Heidelberg University Hospital, Im Neuenheimer Feld 410, 69120 Heidelberg, Germany; 2 Liver Cancer Center Heidelberg LCCH, Im Neuenheimer Feld 460, 69120 Heidelberg, Germany; 3 Department of Medical Oncology, Heidelberg University Hospital, National Center for Tumor Diseases, Im Neuenheimer Feld 460, 69120 Heidelberg, Germany; 4 Department of Diagnostic and Interventional Radiology, University Hospital of Heidelberg, 69120 Heidelberg, Germany; 5 Department of Radiation Oncology, Heidelberg University Hospital, Im Neuenheimer Feld 400, 69120 Heidelberg, Germany; 6 Department of Pathology, Heidelberg University Hospital, Im Neuenheimer Feld 224, 69120 Heidelberg, Germany; 7 Department of Nuclear Medicine, Heidelberg University Hospital, Im Neuenheimer Feld 400, 69120 Heidelberg, Germany; 8 Department of General, Visceral and Transplantation Surgery, Heidelberg University Hospital, Im Neuenheimer Feld 110, 69120 Heidelberg, Germany; 9 Department of Internal Medicine, Salem Hospital Heidelberg, Zeppelinstraße 11-33, 69121 Heidelberg, Germany; 10 Division of Chronic Inflammation and Cancer, German Cancer Research Center Heidelberg (DKFZ), Im Neuenheimer Feld 280, 69120 Heidelberg, Germany

**Keywords:** Hepatocellular carcinoma, Stage migration, Treatment sequence, Liver transplantation, TACE

## Abstract

**Purpose:**

This retrospective analysis focuses on treatment stage migration in patients with hepatocellular carcinoma (HCC) to identify successful treatment sequences in a large cohort of real-world patients.

**Methods:**

1369 HCC patients referred from January 1993 to January 2020 to the tertiary center of the Heidelberg University Hospital, Germany were analyzed for initial and subsequent treatment patterns, and overall survival.

**Results:**

The most common initial treatment was transarterial chemoembolization (TACE, *n* = 455, 39.3%) followed by hepatic resection (*n* = 303, 26.1%) and systemic therapy (*n* = 200, 17.3%), whereas the most common 2nd treatment modality was liver transplantation (*n* = 215, 33.2%) followed by systemic therapy (*n* = 177, 27.3%) and TACE (*n* = 85, 13.1%). Kaplan–Meier analysis revealed by far the best prognosis for liver transplantation recipients (median overall survival not reached), followed by patients with hepatic resection (11.1 years). Patients receiving systemic therapy as their first treatment had the shortest median overall survival (1.7 years; *P* < 0.0001). When three or more treatment sequences preceded liver transplantation, patients had a significant shorter median overall survival (1st seq.: not reached; 2nd seq.: 12.4 years; 3rd seq.: 11.1 years; beyond 3 sequences: 5.5 years; *P* = 0.01).

**Conclusion:**

TACE was the most common initial intervention, whereas liver transplantation was the most frequent 2nd treatment. While liver transplantation and hepatic resection were associated with the best median overall survival, the timing of liver transplantation within the treatment sequence strongly affected median survival.

**Supplementary Information:**

The online version contains supplementary material available at 10.1007/s00432-021-03528-3.

## Introduction

Cancer-related death caused by hepatocellular carcinoma (HCC) is worldwide on the rise, currently accounting for the fifth most prevalent type of cancer and for the third most common cause of cancer-related mortality (Cunha et al. 2020; Lingiah et al. 2020). The broad spectrum of therapeutic interventions ranges from curative options over bridging to curative settings to palliative treatments.

Liver transplantation, hepatic resection and locoregional ablative interventions are regarded as potential curative treatments. TACE has been the most commonly used method to bridge HCC patients to transplantation (Affonso et al. 2019; Kollmann et al. 2017). Within the last decade, the repertoire of systemic therapies has been significantly expanded, from the multi-kinase inhibitors sorafenib and regorafenib, to the vascular endothelial growth factor (VEGF) receptor multi-tyrosine kinase inhibitors lenvantinib and cabozantinib, and therapeutic anti-VEGF-2 antibody ramucirumab, as well as to immune-checkpoint inhibitors such as nivolumab and pembrolizumab, and the combination of atezolizumab and bevacizumab (Finn et al. 2020).

Clinical decision making is guided by interdisciplinary tumor boards, yet selecting the most favorable option, especially in sequential therapies across different disease stages, remains challenging (Allaire and Nault 2017; Kirstein et al. 2020, 2017). Therapeutic stratification of HCC patients is conducted by imaging, clinical criteria and the extent of liver function assessed most commonly by the Barcelona Clinic Liver Cancer (BCLC) classification, the Eastern Cooperative Oncology Group (ECOG) performance status and the Child–Pugh score (Forner et al. 2009; Hinrichs et al. 2017).

However, given the minority of only about 20% of HCC patients who are initially suitable for curative treatment options, the development of concepts that focus on therapeutic sequencing to contain the disease in the most successful manner has become crucial. Along the process of treatment stage migration, the flexibility of the stage hierarchy has strongly increased within the recent years (Vitale et al. 2020). To gain a more detailed picture of survival, and as treatment options, apart from systemic therapy, have essentially remained constant over the last decades, we aimed to evaluate the success of real-world treatment patterns after initial diagnosis and in subsequent therapy modalities in 1369 HCC patients referred to the tertiary center liver cancer unit at Heidelberg University Hospital for the extended period of 27 years.

## Methods

Patients included in this retrospective analysis had a histologically or radiographically confirmed diagnosis of HCC according to EASL guidelines and were 18 years of age or older at the time of data acquisition. Assessment of histology, transient elastography and imaging (CT, MRI, ultrasound) was performed to confirm the diagnosis of cirrhosis. Radiographic data, clinical presentation, and laboratory assessments at the time of the initial treatment for HCC were applied to analyze the cohort of treated patients. In case of repetitive treatments (e.g., multiple TACE) in one sequence, the data set of the first performed procedure was analyzed. Time span of overall survival (OS) was calculated from HCC diagnosis until last follow-up or time point of death. For the analysis of therapeutic sequencing, the order of treatment sequences was defined as the transition from one to another therapeutic option after recurrent or progressive disease, due to intolerable adverse effects, or in case of potentially curative procedures repetition of the same treatment following local or, when limited to the liver, distant recurrence. From 1996 onward a hepatobiliary board was established at our liver center based on which interdisciplinary treatment decisions were drawn. In addition, liver transplantation would be performed whenever feasible following a local interdisciplinary transplantation board review in accordance with national and international transplantation guidelines. Due to incomplete data, 41 patients were excluded from analysis. Treatment options included liver transplantation (LTX), hepatic resection (OP), radiofrequency ablation (RFA), microwave ablation (MWA), percutaneous ethanol injection (PEI), irreversible electroporation (IRE), transarterial chemoembolization (TACE), selective internal radiation therapy (SIRT), stereotactic body radiotherapy (SBRT), and systemic therapy (Systemic). From 2010 onward the results of the PRECISION V trial (Lammer et al. 2010) were incorporated at our liver cancer center and TACE interventions were thenceforth performed with drug-eluting beads (DEB-TACE). Intra-arterial chemoperfusion (degradable starch microspheres) was alternatively performed in patients with impaired liver function. RFA, MWA, PEI and IRE were grouped as ablative interventions (Ablation). By the year 2018, the predominant ablative intervention was MWA replacing RFA, whereas throughout the entire observation period PEI was very restrictively applied. Systemic therapies included sorafenib (since 2007), regorafenib (since 2016), lenvatinib (since 2018), cabozantinib (since 2018), nivolumab (since 2015), pembrolizumab (since 2018), and ramucirumab (since 2014). Within the treatment hierarchy liver transplantation was regarded as the most superior form of therapy, followed from left to right by hepatic resection, ablative interventions, TACE, SIRT, SBRT and systemic therapy.

### Ethical standards

The data acquisition and analysis were a priori approved by the local ethics committee of the medical faculty of Heidelberg and was conducted according to the in 2000 revised ethical standards defined by the Declaration of Helsinki of 1975. The issued ethics approval number S-043/2011.

### Statistical analysis

GraphPad Prism version 5 software (GraphPad Software, San Diego, CA, USA) and Microsoft Excel 365 (Microsoft Corporation, Redmond, WA, USA) were used for statistical and graphical analyses. Data are presented as mean ± standard deviations for continuous variables and as median with interquartile ranges (25%-75%) for discrete variables. Categorical variables were analyzed applying the Fisher’s exact test, whereas continuous variables were analyzed applying the unpaired Student’s *t*-test. The Mantel-Cox log-rank test was applied to assess overall survival using the Kaplan–Meier estimation. A *P*-value < 0.05 was considered statistically significant.

## Results

### Patients` characteristics

Patients diagnosed with HCC analyzed in this study are described in Table 1. A total of 1159 (84.7%) patients received *n* ≥ 1 treatment modality, and 210 (15.3%) patients were without documented treatment until last follow-up. Apart from overrepresentation of viral hepatitis C in patients with *n* ≥ 1 treatment modality, there was no significant difference in etiology between the cohorts. Extrahepatic spread was more common in patients without documented treatment (7.4% vs. 12.4%; *P* = 0.0199). Patients receiving *n* ≥ 1 treatment modality had in comparison to BSC-treated patients lower Child–Pugh scores, ECOG scores and BCLC stages.

**Table 1:** Baseline characteristics of patients diagnosed with HCC treated with n ≥ 1 treatment vs. patients without documented treatment until last follow-up

Patients characteristics	*n* ≥ 1 treatment		Without treatment		*P* value
	Number	Percent (%)	Number	Percent (%)	
Total patients	1159	100	210	100	
*General*					
Sex, male	926	79.9	173	82.4	*ns*
Sex, female	233	20.1	37	17.6	*ns*
Age at HCC diagnosis	61.4 (± 11.0)		66.3 (± 9.9)		< 0.0001
Death	342	29.5	27	12.9	< 0.0001
Metastatic disease	86	7.4	26	12.4	0.0199
Lung metastases	36	3.1	18	8.6	0.0007
Lymph node metastases	32	2.8	12	5.7	0.0328
Bone metastases	13	1.1	6	2.9	*ns*
Other location^#^	12	1.0	4	1.9	*ns*
Clinical status					
Child A	799	68.9	106	50.5	< 0.0001
Child B	259	22.3	78	37.1	< 0.0001
Child C	101	8.7	26	12.4	*ns*
BCLC initial A	332	28.6	19	9.0	< 0.0001
BCLC initial B	402	34.7	40	19.0	< 0.0001
BCLC initial C	339	29.2	122	58.1	< 0.0001
BCLC initial D	43	3.7	26	12.4	< 0.0001
BCLC NA	43	3.7	4	1.9	*ns*
ECOG initial 0	880	75.9	88	41.9	< 0.0001
ECOG initial 1	225	19.4	80	38.1	< 0.0001
ECOG initial 2	26	2.2	28	13.3	< 0.0001
ECOG initial 3–4	5	0.4	13	6.2	< 0.0001
ECOG NA	23	2.0	1	0.5	*ns*
*Liver cirrhosis*					
Yes	889	76.7	160	76.2	*ns*
No	270	23.3	50	23.8	*ns*
*Etiology*					
Alcoholic liver disease	319	27.5	67	31.9	*ns*
Hepatitis B	228	19.7	33	15.7	*ns*
Hepatitis C	336	29.0	44	21.0	0.0188
Non-alcoholic steatohepatitis	29	2.5	4	1.9	*ns*
Autoimmune hepatitis	10	0.9	0	0.0	*ns*
Primary biliary cholangitis	7	0.6	0	0.0	*ns*
Primary sclerosing cholangitis	4	0.3	0	0.0	*ns*
Cryptogenic	73	6.3	9	4.3	*ns*
Other^##^	14	1.2	3	1.4	*ns*

^#^Other location of metastatic disease included adrenal, peritoneal, and pancreatic. ^##^In patients treated with *n* ≥ 1 line of therapy haemochromatosis was diagnosed in 9 (0.8%) patients, 2 (0.2%) patients were included with Wilson's disease and 3 (0.3%) patients had an acute liver failure, whereas haemochromatosis was diagnosed in 3 (1.4%) patients without treatment

### Treatment distribution

**Fig. 1:**
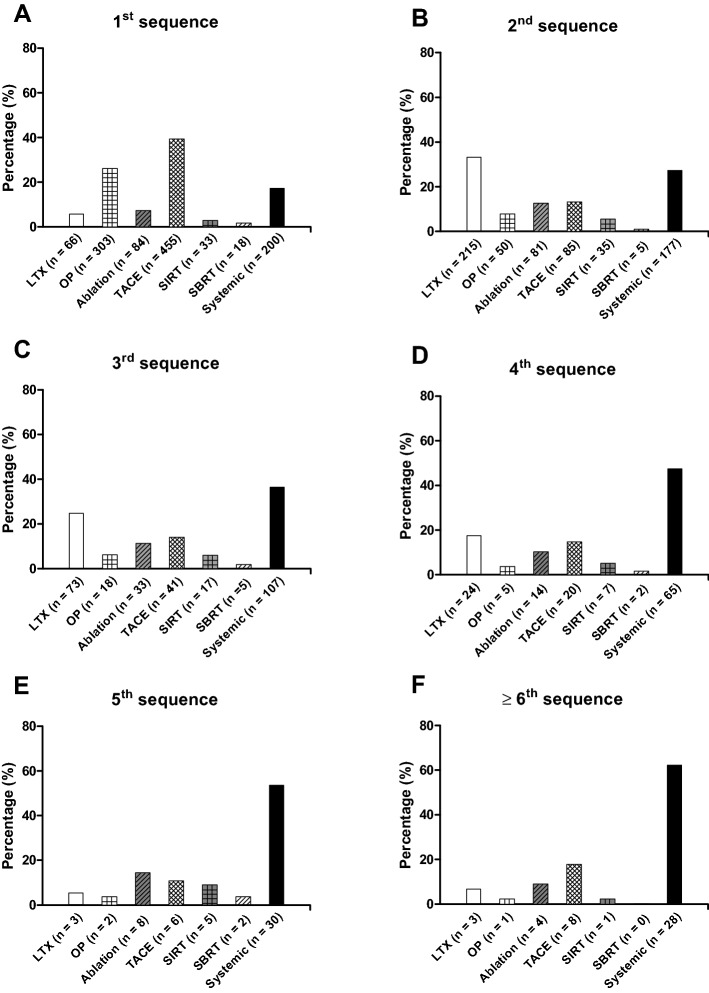
Distribution of treatment modalities among patients receiving *n* ≥ 1 sequence of therapy. **a** The most common 1st treatment sequence was TACE followed by hepatic resection and systemic therapy. **b** The most common 2nd treatment sequence was liver transplantation followed by systemic therapy and TACE. **c**–**f** From the 3rd treatment sequence and beyond systemic therapy was the most common treatment with a steadily increasing percentage. *LTX* liver transplantation, *OP* hepatic resection, *Ablation* ablative procedures including RFA, MWA, IRE and PEI, *TACE* transarterial chemoembolization, *SIRT* selective internal radiation therapy, *SBRT* stereotactic body radiotherapy, *Systemic* systemic therapy

Distribution of treatment modalities among the 1159 patients receiving n ≥ 1 treatment sequence is depicted in Fig. 1. The most common initial treatment was TACE (39.3%) followed by hepatic resection (26.1%) and systemic therapy (17.3%; Fig. 1a), whereas the most common treatment modality as 2nd sequence was liver transplantation (33.2%) followed by systemic therapy (27.3%) and TACE (13.1%; Fig. 1b). From the 3rd sequence and beyond, systemic therapy was increasingly the most common modality (3rd seq.: 36.4%, ≥ 6th seq.: 62.2%; Fig. 1c–f). After admission of Lenvatinib in 2018, Lenvatinib was the most common used systemic therapy in the year 2019 replacing sorafenib (Suppl. Fig. 1a).

The extent of tumor load varied considerably between the initial treatment modalities (Table 2). Patients initially treated with liver transplantation had the lowest mean AFP level (57 ± 239 IU/ml), no extrahepatic spread and the second smallest diameter of the largest tumor (25; 16–35 mm) after patients which had locoregional ablative intervention (20; 14–30 mm). Patients undergoing liver transplantation were also characterized by the highest median Child–Pugh score (8; 6–10). In contrast, patients admitted to systemic treatment as their 1st treatment modality had the highest tumor burden represented by the largest tumor size (70; 40–100 mm) and number (> 3; 3– > 3). In addition, systemically treated patients had the highest mean AFP level (85,411 ± 443,697 IU/ml) and largest fraction of extrahepatic spread of 31.5%. Patients undergoing liver transplantation were youngest with a mean age of 53.7 ± 9.2 years, while patients treated with SBRT were oldest (72.6 ± 8.5 years).

**Table 2:** Distribution of tumor load according to initial treatment modality

Initial treatment	LTX (*n* = 66)	OP (*n* = 303)	Ablation (*n* = 84)	TACE (*n* = 455)	SIRT (*n* = 33)	SBRT (*n* = 18)	Systemic (*n* = 200)
Age (years) Mean (± SD)	53.7 (± 9.2)	60.5 (± 13.0)	61.4 (± 8.6)	61.1 (± 9.5)	67.1 (± 7.5)	72.6 (± 8.5)	64.2 (± 11.0)
AFP (IU/ml) Mean (± SD)	57 (± 239)	4663 (± 37,481)	304 (± 1842)	1267 (± 7493)	414 (± 870)	213 (± 568)	85,411 (± 443,697)
Largest tumor size (mm) Median (IQR 25–75)	25 (16–35)	50 (30–75)	20 (14–30)	33 (23–50)	45 (36–72)	40 (35–44)	70 (40–100)
Tumor number Median (IQR 25–75)	1 (1–2)	1 (1–2)	1 (1–2)	2 (1–> 3)	> 3 (3–> 3)	1 (1–2)	> 3 (3–> 3)
Extrahepatic spread *n* (%)	0 (0)	12 (4.0)	0 (0)	8 (1.8)	2 (6.1)	1 (5.5)	63 (31.5)
CPS Median (IQR 25–75)	8 (6–10)	5 (5–6)	5 (5–6)	6 (5–8)	5 (5–6)	6 (5–7)	6 (5–7)
BCLC Median (IQR 25–75)	B (A–C)	B (A–B)	A (A–B)	B (A–B)	A (A–B)	B (B–C)	C (B–C)

*SD* standard deviation, *IQR* interquartile range, *CPS* Child–Pugh-Score, *LTX* liver transplantation, *OP* hepatic resection, *Ablation* ablative procedures including RFA, IRE and PEI, *TACE* transarterial chemoembolization, *SIRT* selective internal radiation therapy, *SBRT* stereotactic body radiotherapy, *Systemic* systemic therapy

Following BCLC recommendations, in summary most patients categorized as BCLC A received either liver transplantation (6.9%), hepatic resection (29.8%) or a locoregional ablative intervention (16.0%) as their first treatment modality (Table 3). However, TACE was the most common treatment for early- as well as intermediate-staged patients (BCLC A and B; each 43.4%). From 2008 onwards, TACE was the most common intervention performed in our cohort (Suppl. Fig. 1b). Overall, only in a minority of 2.0% of all performed TACE interventions the patients had a concomitant drug treatment. Both SIRT and SBRT were rarely chosen as treatment options. Most patients with the advanced BCLC stage C underwent systemic treatment (47.5%) and within the group of systemically treated patients, the majority was advanced staged (70.0%). Liver transplantation (58.3%) was the most frequent treatment in BCLC stage D patients followed by TACE (33.3%).

**Table 3:**
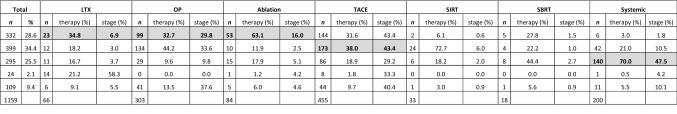
Distribution of BCLC classification according to initial treatment modality (numbers in bold display treatments within the recommended BCLC stage)

*LTX* liver transplantation, *OP* hepatic resection, *Ablation* ablative procedures including RFA, MWA, IRE and PEI, *TACE* transarterial chemoembolization, *SIRT* selective internal radiation therapy, *SBRT* stereotactic body radiotherapy, *Systemic* systemic therapy

### Stage migration

Subsequent stage migration following 1st line therapy to treatment modalities indicated for more advanced disease stages was documented in 60.2% of the treatment choices, whereas 39.8% of the subsequent interventions were indicated for the same or earlier disease stages (Table 4). Most patients did not undergo any further interventions following liver transplantation (81.8%), SBRT (83.3%) and systemic treatment (75.5%). However, only about one-third of the patients treated by TACE (33.0%), liver resection (39.3%) and locoregional ablative intervention (40.5%) did not receive any subsequent treatment. In the following course 42.0% of patients treated initially with TACE, 34.5% of patients with locoregional ablative interventions and 10.6% of liver resected patients would be transplanted. In contrast, only a minority of patients with SBRT (5.6%) or systemic treatment (1%), and no patient with SIRT would undergo liver transplantation in subsequent treatments.

**Table 4:**
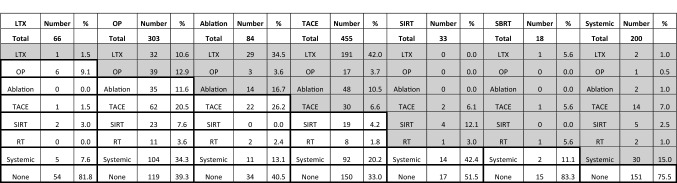
Stage migration beyond 1^st^ treatment sequence. Bold font indicates the chosen initial intervention

*LTX* liver transplantation; *OP* hepatic resection; Ablation, ablative procedures including RFA, MWA, IRE and PEI; *TACE* transarterial chemoembolization; *SIRT* selective internal radiation therapy; *SBRT* stereotactic body radiotherapy; *Systemic* systemic therapy

Bold frames display subsequent treatment modalities in accordance with stage migration. Gray background highlights subsequent interventions which are indicated for the same or earlier disease stages.

### Survival analysis

**Fig. 2:**
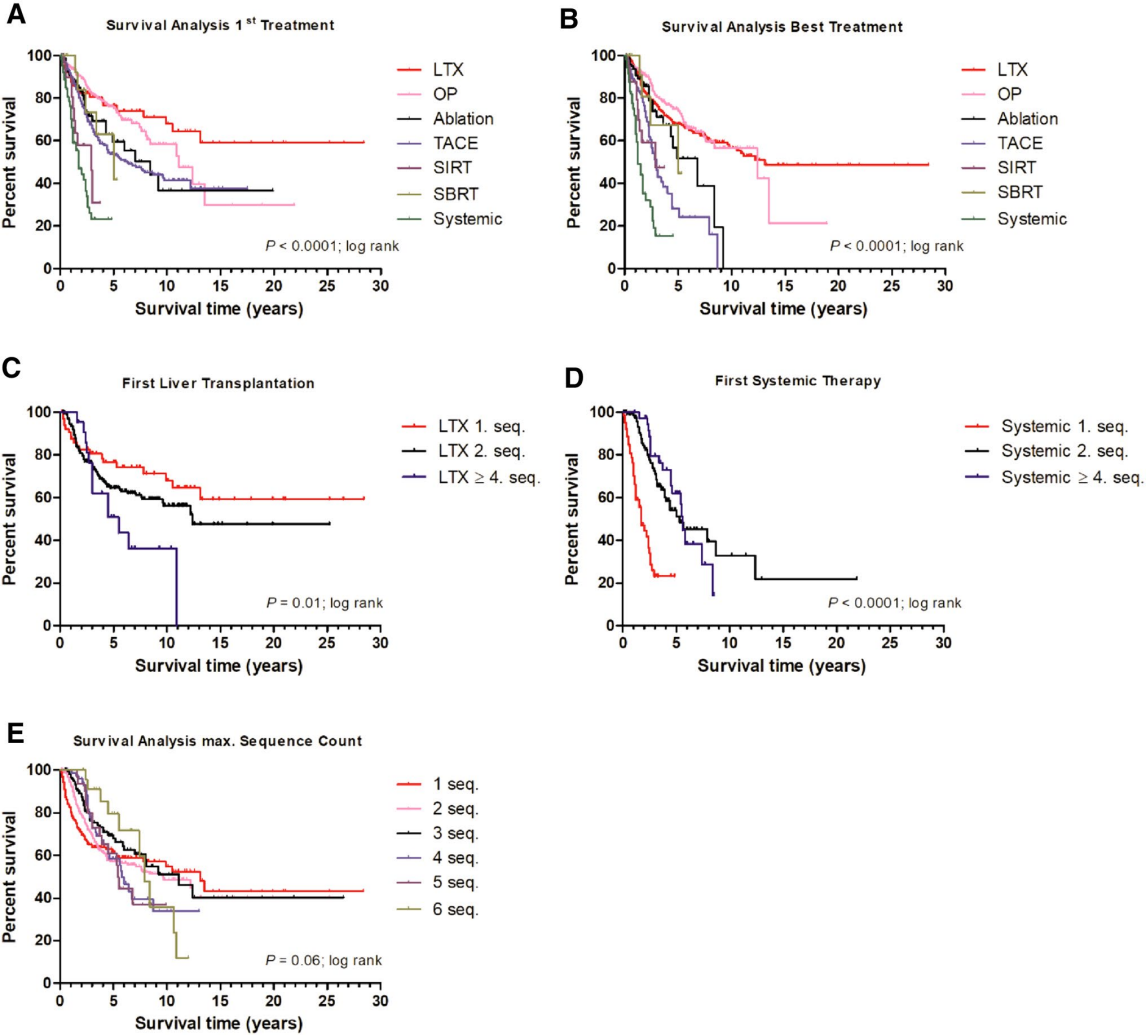
Kaplan–Meier survival analysis of the studied HCC patients according to 1st treatment modality (**a**), overall best treatment modality according to the treatment hierarchy (**b**), the best achieved treatment sequence of the first liver transplantation (**c**) and first systemic therapy (**d**), and maximum count of sequences (**e**). **a** and **b** Liver transplantation and hepatic resection were associated with longest median survival, whereas systemically treated patients had the shortest median survival both for the 1st treatment line and best treatment modality. **c** Patients transplanted as their 1st treatment had a better mOS compared to patients transplanted in 2nd sequence and beyond the 3rd sequence of treatment (1st seq., *n* = 66, median overall survival not reached; 2nd seq., *n* = 206, 12.4 years; ≥ 4th seq., *n* = 22, 5.5 years). **d** Patients subjected to systemic treatment as their 1st treatment had a shorter mOS compared to 2nd sequence and beyond their 3rd sequence of treatment (1st seq., *n *= 200, 1.7 years; 2nd seq., *n* = 149, 5.3 years; ≥ 4th seq., *n* = 38, 5.6 years). **e** Patients treated with only one treatment sequence had within the first years a poorer prognosis compared to patients treated up to their 6th sequence, however, on the long-term, median survival was inferior for patients with more than one treatment sequence. *LTX* liver transplantation, *OP* hepatic resection, *Ablation* ablative procedures including RFA, MWA, IRE and PEI, *TACE* transarterial chemoembolization, *SIRT* selective internal radiation therapy, *SBRT* stereotactic body radiotherapy, *Systemic* systemic therapy, *mOS* median overall survival, *seq.* sequence

Kaplan–Meier survival analysis of the studied HCC patients according to 1^st^ treatment modality revealed longest overall survival (OS) for transplanted patients, where the median OS was not reached (Fig. 2a, Suppl. Fig 2a–f). Patients with liver resection had a median OS of 11.1 years, followed by patients who underwent locoregional ablative intervention with 8.4 years. Median OS for patients treated with TACE as 1st treatment modality was 6.3 years, whereas patients treated with SIRT and SBRT had a median OS of 2.9 and 5.5 years, respectively. The shortest median OS with 1.7 years was obtained in patients receiving systemic therapy as initial treatment.

When analyzed for best achieved treatment according to defined treatment hierarchy, patients with liver transplantation had the most favorable outcome with a median OS of 13.1 years followed by liver resected patients with a median OS of 12.4 years, and then by locoregional ablative intervention with a median OS of 6.8 years (Fig. 2b, Suppl. Fig. 3a–f). Patients with TACE as the best achieved treatment had a median OS of 3.0 years, whereas again the shortest median OS was seen in patients under systemic treatment with 1.2 years.

Patients with liver transplantation had a significant worse median OS if they were transplanted beyond their 3^rd^ treatment sequence compared to the 1st sequence (Fig. 2c; 1st seq.: median OS not reached; 2nd seq.: 12.4 years; 3rd seq.: 11.1 years; beyond 3rd treatment sequence: 5.5 years; *P* = 0.01). In contrast, patients subjected to their first systemic treatment beyond the 3rd treatment sequence had a significant longer median OS (Fig. 2d; 1st seq.: median OS 1.7 years; 2nd seq.: 5.3 years; 3rd seq.: 4.0 years; beyond 3rd treatment sequence: 5.6 years; *P* < 0.0001). A similar trend of longer median OS was seen for all other modalities except for hepatic resection, which was firstly performed only in one patient beyond 3rd treatment sequence.

More than half of the patients received a subsequent therapy following initial treatment (648 patients; 55.9%). One third of the patients received up to two sequences of treatment (354 patients; 30.5%), whereas a total of 157 (13.5%) and 81 (6.9%) patients received a 3rd and 4th subsequent treatment, respectively. A 5th treatment sequence was achieved by 30 (2.6%) and up to and beyond 6^th^ treatment sequence was documented in 24 (2.1%) patients. Patients treated with a single treatment modality had within the first years a poorer prognosis compared to patients treated up to their 6th treatment sequence. However, on the long-term, median OS was higher in patients with fewer treatment sequences (Fig. 2e, Suppl. Fig. 4a–e; 1st seq.: 13.1 years; 2nd seq.: 9.7 years; 3rd seq.: 11.1 years) compared to patients with more than three treatment sequences (4th seq.: 5.8 years; 5th seq.: 5.4 years; 6th seq.: 7.9 years). Patients treated with TACE at an early or intermediate BCLC stage had irrespective of the treatment modality a significant better median OS compared to patients classified as BCLC C or D (Suppl. Fig. 5a, b; BCLC A/B, 7.1 years; BCLC C/D, 4.5 years; *P* < 0.01).

## Discussion

In this retrospective study 1369 patients affected with HCC attending from January 1993 to January 2020 a tertiary center liver cancer unit were analyzed regarding baseline characteristics and subsequent treatment algorithms. Our real-world population displays the strong enrichment of the spectrum of therapeutic options and of treatment sequences beyond 1^st^ line of therapy that has evolved over the last decade with emerging systemic treatments opening options to patients who otherwise would have been limited to best supportive care.

TACE was the most common initial treatment modality in consistency with previously published studies (Akada et al. 2019; Hong et al. 2016; Kirstein et al. 2017; Park et al. 2015). Two-thirds (67.0%) of the patients were retreated after initial TACE compared to 73.9% in a Korean cohort (Hong et al. 2016) and 62.4% in a German cohort (Kirstein et al. 2017), whereas subsequent treatments were less common in patients other than TACE as first treatment. Performance of TACE beyond BCLC stage B was associated with a worse median overall survival, which did not depend on the treatment sequence. Patients initially treated by TACE had a relatively long median overall survival of 6.3 years compared to 10.3–25.4 months in other cohorts (Kirstein et al. 2017; Ogasawara et al. 2020; Yang et al. 2020). This observation can be partially explained by the much higher proportion of patients treated initially with TACE (42.0%) in our cohort undergoing subsequent liver transplantation, as compared to for example the cohort described by Kirstein et al. (8.1%). Throughout all BCLC stages, TACE was more often performed and the median size of the largest tumor (33; 23-50 mm) and median tumor number (2; 1- > 3) were smaller in our cohort compared to Kirstein et al. with 50 mm (34–75 mm) and 3 (1– > 3), respectively (Kirstein et al. 2017). In alignment with Kishore et al. TACE was also in our cohort the most common intervention to bridge to transplant (Kishore et al. 2020), followed by local ablative interventions with 34.5% and surgical resection with 10.6% of the respective patients being transplanted in subsequent treatment lines.

Liver transplantation is recognized as the gold standard for the treatment of HCC within the Milan criteria (Orcutt and Anaya 2018). In Germany, malignancy represents the 3^rd^ most common cause of liver transplantation (Tacke et al. 2016). In contrast to RFA in the cohort described by Kirstein et al. in 2017, liver transplantation was in our studied HCC population the most common 2nd treatment sequence. In consistency with previously published studies, less than 20% of the transplanted patients in our cohort received subsequent treatment due to recurrence of disease (Kim et al. 2020; Kirstein et al. 2017; Ramanathan et al. 2014). Furthermore, liver transplantation had the best prognosis, not reaching median overall survival when performed as first therapeutic sequence. This stresses the crucial role of liver transplantation, for which comparable 5-year post-transplant survival and recurrence-free probabilities were achieved, when the response accomplished by downstaging was able to meet the Milan criteria (Couri and Pillai 2019; Lingiah et al. 2020; Yao et al. 2015). Median overall survival remained above 10 years when first liver transplantation is performed up to the 3rd treatment sequence, yet there is an abrupt and significant reduction to 5.5 years when transplantation is performed beyond the 3rd sequence. This significant reduction of survival should be critically evaluated regarding the chronic shortage of donor organs (Lucidi et al. 2015; Pagano et al. 2020).

Patients with hepatic resection had the second-best prognosis with a median overall survival of 11.1 years when performed as initial treatment. In line with treatment recommendations and previously published trials, this group was characterized by a median size of 50 mm large, mostly solitary tumors, and liver function was well preserved with a median Child–Pugh score of 5 points (Chen et al. 2006; Kirstein et al. 2017; Orcutt and Anaya 2018). Locoregional ablative interventions were mostly performed on solitary tumors with a median size of 20 mm in patients with an equally preserved liver function. Due to the less invasive nature of ablative interventions, they have been shown to be associated with fewer complications and shorter hospital stays (Orcutt and Anaya 2018; Wang et al. 2014).

Although limited by a small number, SBRT had a comparable overall survival with TACE despite their more advanced disease stage represented by a larger median tumor size and larger percentage of extrahepatic spread of 5.5% compared to 1.8% in the TACE group. Interestingly, most patients did not undergo any further treatment following SBRT (83.3%), whereas after TACE 67% of patients needed further therapies. This could be explained by the fact that patients receiving SBRT were older and had mostly only a singular liver lesion. Within the recent years, radiotherapy emerged as a less invasive alternative with a favorable low complication rate and preserving effect on the course of quality of life in HCC patients (Mutsaers et al. 2017; Wehling et al. 2019). SBRT has been proposed as a safe and potentially curative treatment option for HCC, especially for inoperable patients and requires further research (Nakano et al. 2018; Shibuya et al. 2018). In addition, it is considered a safe alternative to conventional bridging approaches prior to transplantation (Sapisochin et al. 2017).

With 17.1% of all initial treatment modalities, our proportion of systemic therapy maintained unchanged compared to the cohort of Kirstein et al. and lies within the range of the BRIDGE trial (Kirstein et al. 2017; Park et al. 2015). In alignment with a recently published sequence analysis of systemic therapies, the most common 1^st^ line treatment was sorafenib with 83.5% (Kirstein et al. 2020). However, this might change with the combinatory treatment of atezolizumab and bevacizumab (Finn et al. 2020). The use of emerging agents other than sorafenib and lenvatinib was continuously increasing in subsequent lines of treatment, reaching more than half of the patients within the 4^th^ treatment sequence and beyond.

A minority of 15.3% did not receive any intervention beyond best supportive care, representing a smaller fraction compared to 23.3% in the cohort described by Kirstein et al., yet similar to the fractions reported in the BRIDGE study for North America and Europe with graphically estimated approximately 16% and 11%, respectively (Kirstein et al. 2017; Park et al. 2015).

The retrospective nature is one limitation of our study, yet it portrays a large real-world cohort over a long treatment period in which treatment algorithms have been improved. Altogether, less than half of all patients were initially treated in line with BCLC stage recommendation. Subsequent stage migration according to the BCLC system for more advanced disease stages occurred in the majority of 60.2% of the treatment choices. However, this also reflects the observations many clinicians share, how in daily routine, treatment choices are commonly made beyond official recommendations. Previously published studies report that 29–51.3% of all patients were treated outside official guidelines (Kirstein et al. 2017; Sangiovanni and Colombo 2016). Nevertheless, we see improvement of survival rates over the last decades as others (Hong et al. 2016), which might be biased in our cohort by a relatively high transplantation rate. The landscape of treatment sequencing has broadened as well as the variety of therapeutic options especially for systemic treatments and the combination of these locoregional interventions, yet the best sequencing strategy remains unclear (Rimassa and Worns 2020). We believe that a systematic treatment approach guided by the establishment of an interdisciplinary hepatobiliary board at our liver center from 1996 onward, which was further strengthened by the foundation of the National Center of Tumor Diseases in Heidelberg in 2004, lead to an improvement of the multimodality treatment algorithm. The multimodality approach as represented also in our cohort appears to result in superior overall survival in contrast to strict adherence to treatment sequences and is well tolerated in most recurrent HCC patients (Fields et al. 2017).

## Supplementary Information

Below is the link to the electronic supplementary material.

Supplementary file1 (DOCX 1364 KB)

## Abbreviations

Ablation: Ablative procedures
AFP: Alpha-fetoprotein
BCLC: Clinic liver cancer classification
BRIDGE trial: Bridge to better outcomes in HCC
BSC: Best supportive care
CPS: Child–pugh-score
CT: Computer tomography
EASL: European association for the study of the liver
ECOG: Eastern cooperative oncology group
Fig.: Figure
HCC: Hepatocellular carcinoma
IRE: Irreversible electroporation
LTX: Liver transplantation
mOS: Median overall survival
MRI: Magnetic resonance imaging
MWA: Microwave ablation
NA: Not available
OP: Hepatic resection
PEI: Percutaneous ethanol injection
RFA: Radiofrequency ablation
SBRT: Stereotactic body radiotherapy
seq.: Sequence
SIRT: Selective internal radiation therapy
Suppl.: Supplementary
Systemic: Systemic therapy
TACE: Transarterial chemoembolization
VEGF: Vascular endothelial growth factor
