# Supplementary material for: Treatment stage migration and treatment sequences in patients with hepatocellular carcinoma: drawbacks and opportunities
Source: J Cancer Res Clin Oncol. 2021 Feb 4;147(8):2471–81. doi: 10.1007/s00432-021-03528-3 (PMC8236446; doi:10.1007/s00432-021-03528-3)
Supplement: Supplementary file 1 — Supplementary file1 (DOCX 1364 KB) [file 432_2021_3528_MOESM1_ESM.docx]

**
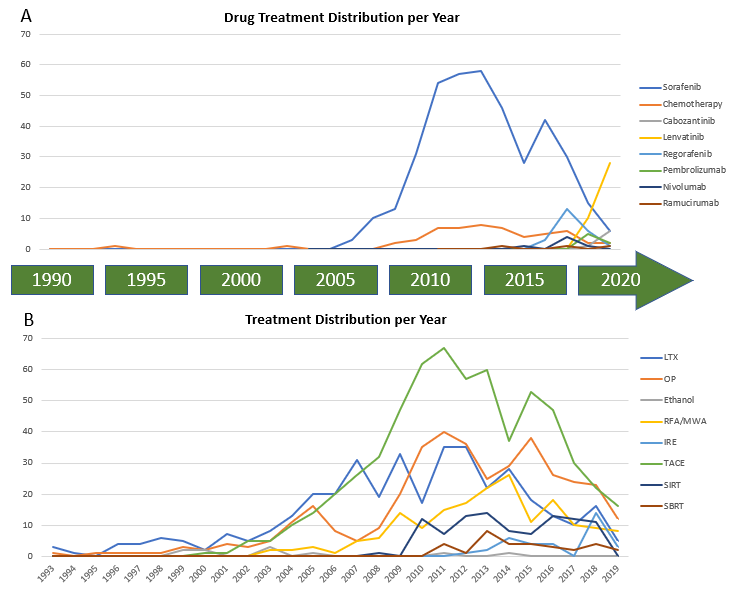
**

**Suppl. Figure 1:** Distribution of treatment modalities according to the year they were performed. Y-axis displays the absolute number of treatments. One patient may be reflected in more than one treatment modality performed in one year. Figure 2a displays the drug spectrum within the treatment modality of systemic therapy. Figure 2B shows the distribution of interventional procedures. LTX, liver transplantation; OP, hepatic resection; RFA, radiofrequency ablation; IRE, irreversible electroporation; MWA, microwave ablation; Ethanol, percutaneous ethanol injection; TACE, transarterial chemoembolization; SIRT selective internal radiation therapy; SBRT, stereotactic body radiotherapy.

**
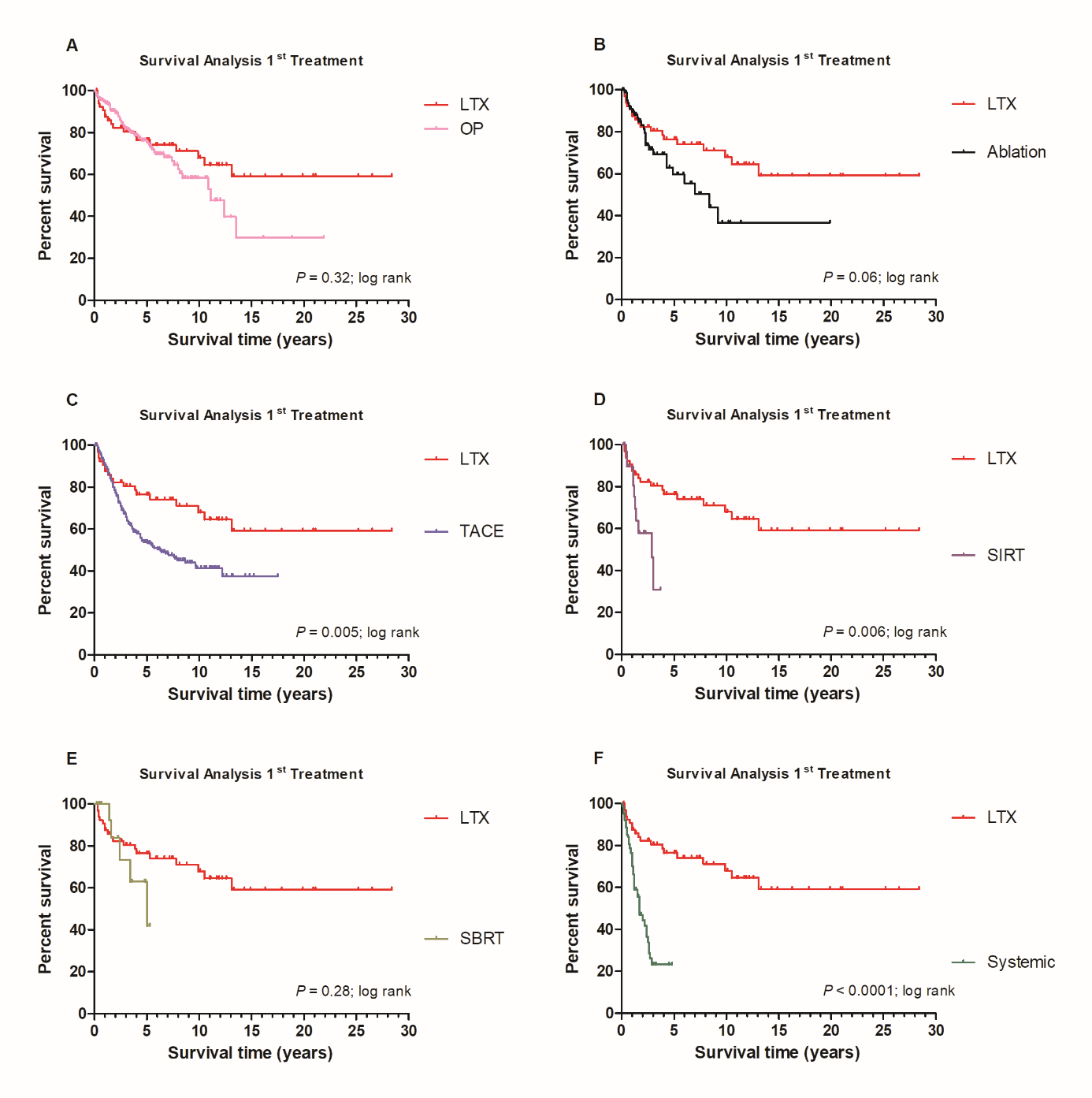
Suppl. Figure 2:** Figures (A)-(F) show the Kaplan–Meier survival analysis of HCC patients according to 1^st^ treatment modality compared to the gold standard of liver transplantation. A: Patients treated with LTX compared to OP (LTX, n = 66, median overall survival not reached; OP, n = 303, median survival 11.1 years). B: Patients treated with Ablation (Ablation, n = 84, 8.4 years). C: Patients treated with TACE (TACE, n = 455, 6.3 years). D: Patients treated with SIRT (SIRT, n = 33, 2.9 years). E: Patients treated with SBRT (SBRT, n = 18, 5.5 years). F: Patients treated with systemic therapy (Systemic, n = 200, 1.7 years). LTX, liver transplantation; OP, hepatic resection; Ablation, ablative procedures including RFA, MWA, IRE and PEI; TACE, transarterial chemoembolization; SIRT selective internal radiation therapy; SBRT, stereotactic body radiotherapy; Systemic, systemic therapy.

**
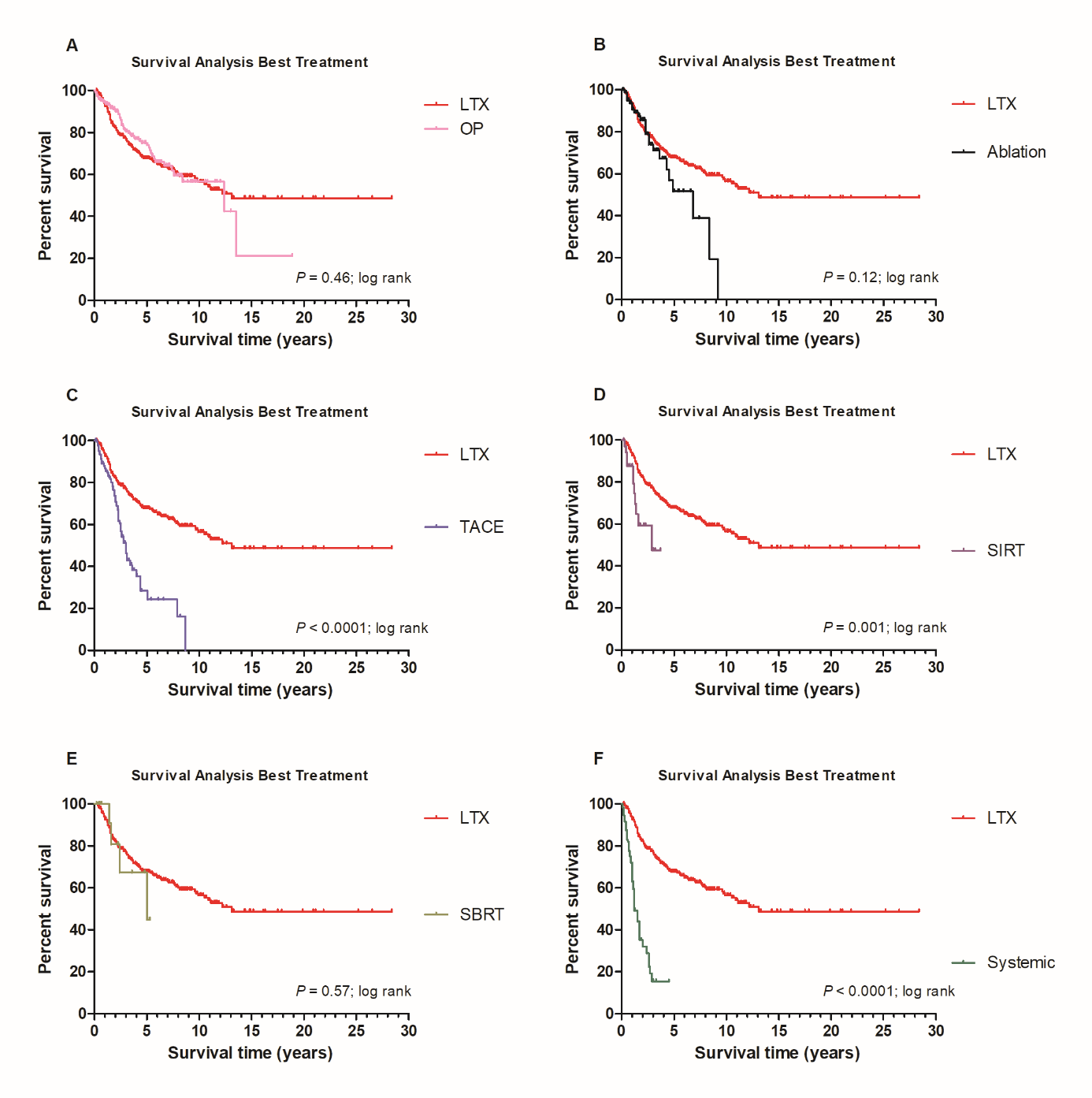
Suppl. Figure 3:** Figures (A)-(F) show the Kaplan–Meier survival analysis of HCC patients according to the best achieved treatment according to the treatment hierarchy compared to the gold standard of liver transplantation. A: Patients treated with LTX compared to OP (LTX, n = 315, median survival 13.1 years; OP, n = 289, median survival 12.4 years). B: Patients treated with Ablation (Ablation, n = 84, 6.8 years). C: Patients treated with TACE (TACE, n = 241, 3.0 years). D: Patients treated with SIRT (SIRT, n = 36, 2.9 years). E: Patients treated with SBRT (SBRT, n = 18, 5.5 years). F: Patients treated with systemic therapy (Systemic, n = 176, 1.2 years). LTX, liver transplantation; OP, hepatic resection; Ablation, ablative procedures including RFA, MWA, IRE and PEI; TACE, transarterial chemoembolization; SIRT selective internal radiation therapy; SBRT, stereotactic body radiotherapy; Systemic, systemic therapy.

**
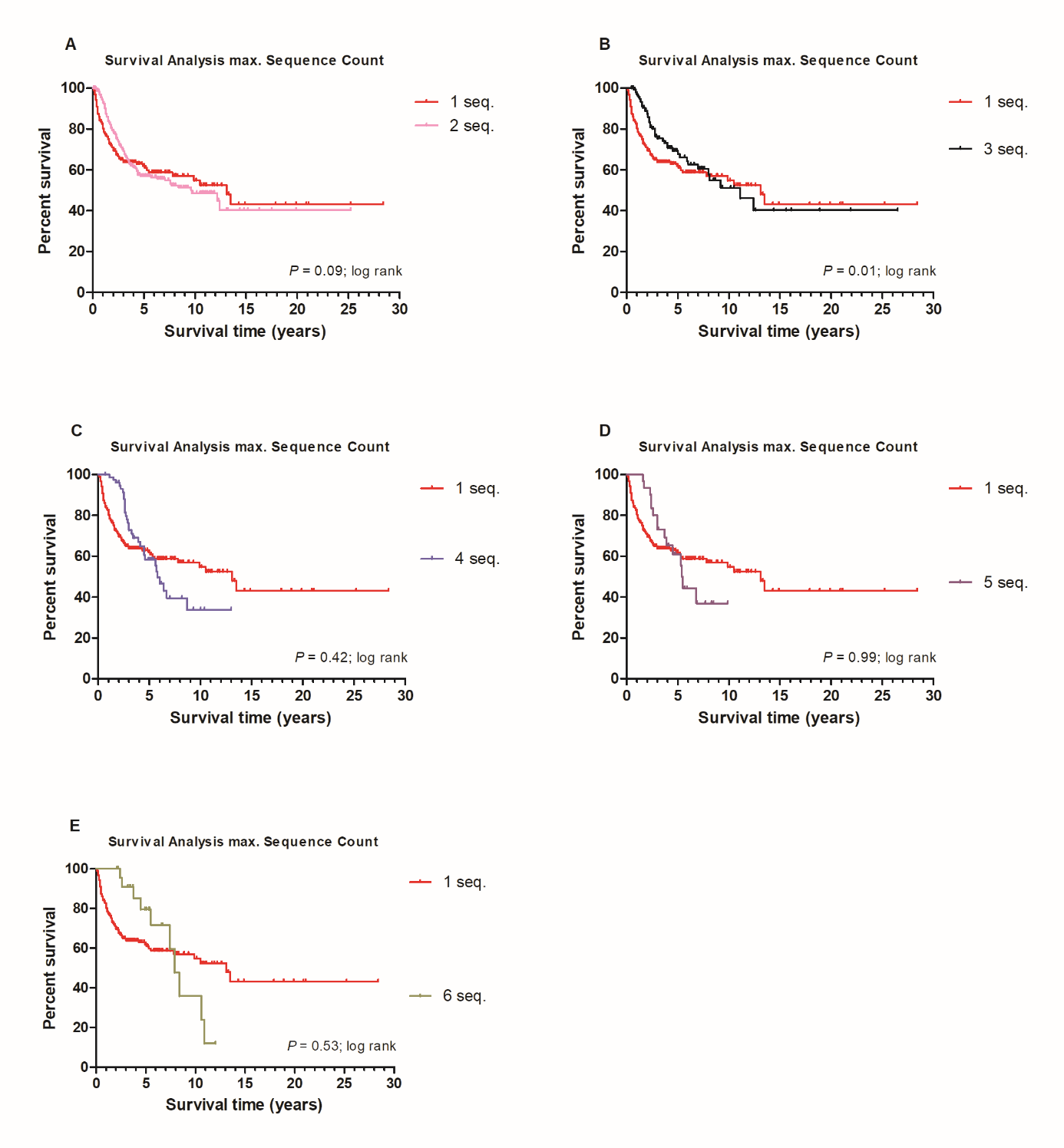
Suppl. Figure 4:** Figures (A)-(E) show the Kaplan–Meier survival analysis of HCC patients according to the best achieved treatment sequence compared to the 1^st^ sequence. A: Patients treated with only one sequence compared to patients received 2 sequences (1 seq., n = 549, median overall survival 13.1 years; 2 seq., n = 368, 9.7 years). B: Patients treated with up to 3 treatment sequences (3 seq., n = 159, 11.1 years). C: Patients treated with up to 4 treatment sequences (4 seq., n = 80, 5.8 years). D: Patients treated with up to 5 treatment sequences (5 seq., n = 31, 5.4 years). F: Patients treated up to 6 treatment sequences (6 seq., n = 24, 7.9 years). Seq., sequence.

**
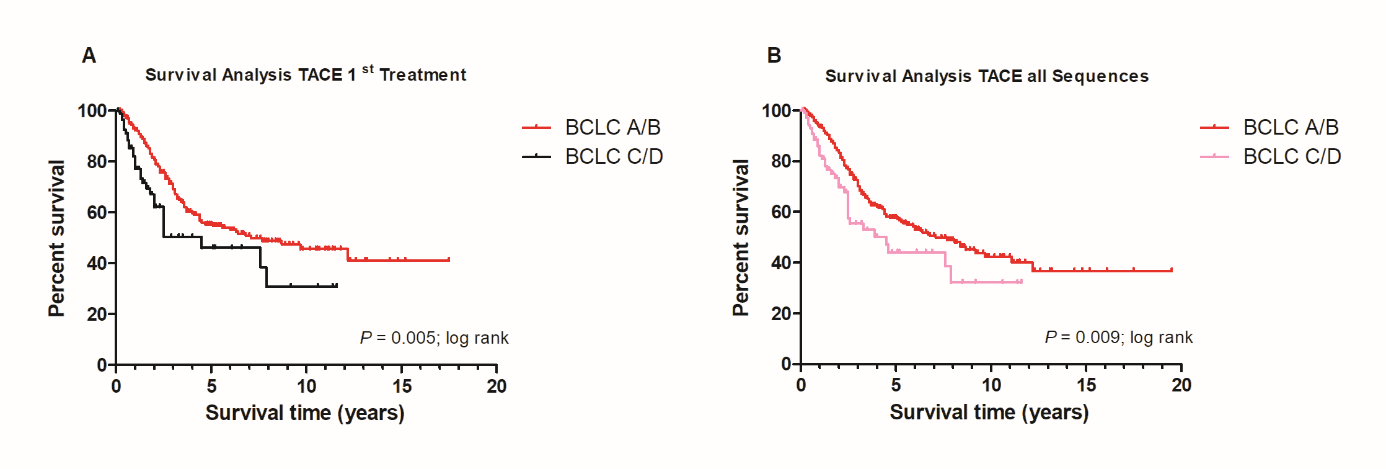
Suppl. Figure 5:** Kaplan–Meier survival analysis of HCC patients with TACE according to BCLC A/B vs. BCLC C/D. Patients classified as BCLC A/B treated with TACE as their 1^st^ treatment (A: BCLC A/B, n = 318, median survival 7.1 years; BCLC C/D, n = 94, 4.5 years) had as well as throughout all sequences (B: BCLC A/B, n = 386, 7.1 years; BCLC C/D, n = 114, 4.5 years) a significant higher median survival rate.

**Suppl. Table 1:** Patients at risk of Figure 2A and Suppl. Figure 2A-F.

| Survival years | LTX | OP | Ablation | TACE | SIRT | SBRT | Systemic |
| --- | --- | --- | --- | --- | --- | --- | --- |
| 0 | 66 | 301 | 84 | 455 | 33 | 18 | 198 |
| 1 | 57 | 242 | 64 | 344 | 20 | 13 | 61 |
| 2 | 47 | 181 | 46 | 234 | 9 | 10 | 18 |
| 3 | 44 | 145 | 30 | 164 | 3 | 7 | 6 |
| 4 | 39 | 112 | 24 | 122 | 0 | 5 | 2 |
| 5 | 35 | 81 | 17 | 100 | 0 | 3 | 0 |
| 10 | 22 | 15 | 4 | 28 | 0 | 0 | 0 |
| 15 | 9 | 3 | 1 | 2 | 0 | 0 | 0 |
| 20 | 6 | 1 | 1 | 0 | 0 | 0 | 0 |
| 25 | 3 | 0 | 0 | 0 | 0 | 0 | 0 |

**Suppl. Table 2:** Patients at risk of Figure 2B and Suppl. Figure 3A-F.

| Survival years | LTX | OP | Ablation | TACE | SIRT | SBRT | Systemic |
| --- | --- | --- | --- | --- | --- | --- | --- |
| 0 | 315 | 287 | 84 | 241 | 36 | 16 | 174 |
| 1 | 290 | 227 | 64 | 145 | 22 | 11 | 41 |
| 2 | 230 | 167 | 41 | 79 | 9 | 8 | 11 |
| 3 | 202 | 129 | 26 | 31 | 3 | 5 | 3 |
| 4 | 176 | 95 | 15 | 13 | 0 | 4 | 1 |
| 5 | 149 | 68 | 9 | 7 | 0 | 3 | 0 |
| 10 | 57 | 10 | 0 | 0 | 0 | 0 | 0 |
| 15 | 13 | 1 | 0 | 0 | 0 | 0 | 0 |
| 20 | 8 | 0 | 0 | 0 | 0 | 0 | 0 |
| 25 | 3 | 0 | 0 | 0 | 0 | 0 | 0 |

**Suppl. Table 3:** Patients at risk of Figure 2C.

| Survival years | LTX 1. Sequ. | LTX 2. Sequ. | LTX ≥ 4. Sequ. |
| --- | --- | --- | --- |
| 0 | 66 | 204 | 22 |
| 1 | 57 | 189 | 22 |
| 2 | 47 | 144 | 20 |
| 3 | 44 | 127 | 16 |
| 4 | 39 | 108 | 11 |
| 5 | 35 | 92 | 8 |
| 10 | 21 | 32 | 2 |
| 15 | 8 | 4 | 0 |
| 20 | 5 | 2 | 0 |
| 25 | 3 | 1 | 0 |

**Suppl. Table 4:** Patients at risk of Figure 2D.

| Survival years | Syst 1. Sequ. | Syst 2. Sequ. | Syst ≥ 4. Sequ. |
| --- | --- | --- | --- |
| 0 | 198 | 149 | 38 |
| 1 | 61 | 124 | 38 |
| 2 | 18 | 82 | 36 |
| 3 | 6 | 53 | 26 |
| 4 | 2 | 34 | 21 |
| 5 | 0 | 19 | 16 |
| 10 | 0 | 5 | 0 |
| 15 | 0 | 1 | 0 |
| 20 | 0 | 1 | 0 |
| 25 | 0 | 0 | 0 |

**Suppl. Table 5:** Patients at risk of Figure 2E and Suppl. Figure 4A-E.

| Survival years | 1 seq. | 2 seq. | 3 seq. | 4 seq. | 5 seq. | 6 seq. |
| --- | --- | --- | --- | --- | --- | --- |
| 0 | 545 | 366 | 159 | 80 | 31 | 24 |
| 1 | 262 | 305 | 146 | 79 | 31 | 24 |
| 2 | 148 | 203 | 116 | 64 | 28 | 24 |
| 3 | 109 | 159 | 77 | 43 | 23 | 20 |
| 4 | 82 | 121 | 62 | 32 | 17 | 15 |
| 5 | 65 | 99 | 42 | 25 | 11 | 13 |
| 10 | 24 | 31 | 12 | 3 | 0 | 3 |
| 15 | 7 | 5 | 5 | 0 | 0 | 0 |
| 20 | 4 | 1 | 2 | 0 | 0 | 0 |
| 25 | 2 | 1 | 1 | 0 | 0 | 0 |

**Suppl. Table 6:** Patients at risk of Suppl. Figure 5A-B.

| Survival years | TACE 1. Seq. BCLC A/B | TACE 1. Seq. BCLC C/D | TACE all Seq. BCLC A/B | TACE all Seq. BCLC C/D |
| --- | --- | --- | --- | --- |
| 0 | 317 | 93 | 386 | 114 |
| 1 | 255 | 50 | 321 | 68 |
| 2 | 185 | 27 | 243 | 41 |
| 3 | 136 | 15 | 180 | 24 |
| 4 | 100 | 13 | 135 | 18 |
| 5 | 82 | 10 | 109 | 13 |
| 10 | 23 | 3 | 26 | 3 |
| 15 | 2 | 0 | 4 | 0 |
| 20 | 0 | 0 | 0 | 0 |
| 25 | 0 | 0 | 0 | 0 |
